# A Case of Pancreatic Metastasis From Small Cell Neuroendocrine Carcinoma of the Oropharynx

**DOI:** 10.7759/cureus.27872

**Published:** 2022-08-11

**Authors:** Shingo Yasutake, Daisuke Mizokami, Saki Takihata, Koji Araki, Akihiro Shiotani

**Affiliations:** 1 Otolaryngology - Head and Neck Surgery, National Defense Medical College, Tokorozawa, JPN; 2 Otolaryngology - Head and Neck Surgery, Nishisaitama Chuo National Hospital, Tokorozawa, JPN; 3 Otolaryngology, Nishisaitama Chuo National Hospital, Tokorozawa, JPN

**Keywords:** bile duct drainage, pancreatic metastasis, head and neck cancer, small cell neuroendocrine carcinoma, extrapulmonary small cell carcinoma

## Abstract

Small cell neuroendocrine carcinoma (SNEC) rarely occurs in the head and neck and usually occurs in the lungs. We report the case of a 55-year-old Asian male with SNEC in the oropharynx and jaundice due to pancreatic metastasis, which was successfully palliated by amrubicin (AMR), radiotherapy, and an endoscopic biliary stent. Although pancreatic metastases are known to occur at the end stage of small cell lung cancer, there are limited data on the treatment protocols for pancreatic metastases from SNEC. The main complication of SNEC for pancreatic lesions is obstructive jaundice. Palliative radiotherapy and biliary drainage may have life-prolonging effects in patients with extrahepatic biliary obstruction. It may also be a worthwhile risk to use anticancer drugs, such as AMR that are metabolized in the liver, if the obstructive jaundice is caused by tumor growth.

## Introduction

Extrapulmonary small cell carcinoma (EPSCC) is a rare aggressive neoplasm that lacks a recommended definitive treatment. Commonly utilized treatments are derived from those for small-cell lung cancer (SCLC) [1]. Pancreatic metastases are extremely rare and occur in the end stage of SCLC. However, few treatment protocols have been reported, even in SCLC. We report a rare case of a patient with pancreatic metastasis from EPSCC in the oropharynx that was successfully treated with salvage chemotherapy, radiation therapy, and bile duct drainage.

## Case presentation

A 55-year-old Asian man was diagnosed with T2N2aM0 stage IVA small cell neuroendocrine carcinoma (SNEC) in the left palatine tonsil, with cervical node metastasis two years prior. Human papillomavirus status was negative on p16 immunohistochemistry. The lesions in the pharynx and neck were reduced after four courses of cisplatin + etoposide, but a follow-up PET scan revealed distant metastasis to the ilium. Three courses of nivolumab were administered; however, the number of tumors in the pharynx and neck increased. After two courses of amrubicin (AMR) with 40 Gy/12 fractions of irradiation on the neck, tumors of the oropharynx and cervical metastasis reduced remarkably. However, owing to the adverse events of febrile neutropenia and severe stomatitis, chemotherapy could not be administered for two months.

**Figure 1:**
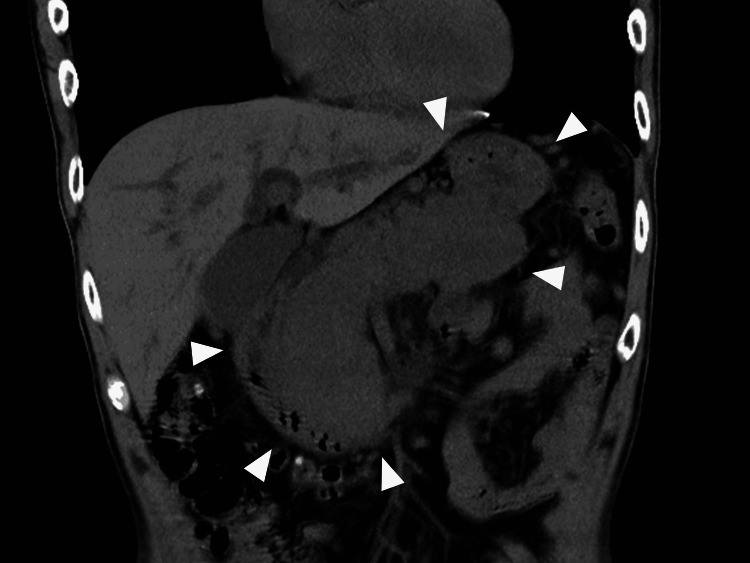
The pancreas (arrowhead) was enlarged due to metastasis.

**Figure 2:**
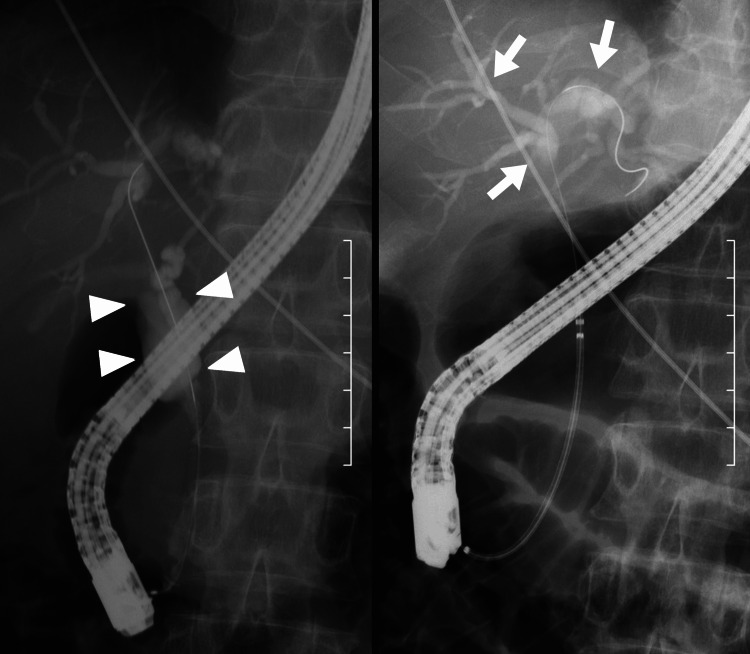
Endoscopic retrograde cholangiopancreatography revealed a markedly dilated common bile duct (arrowhead) and intrahepatic biliary ducts (arrow).

**Figure 3:**
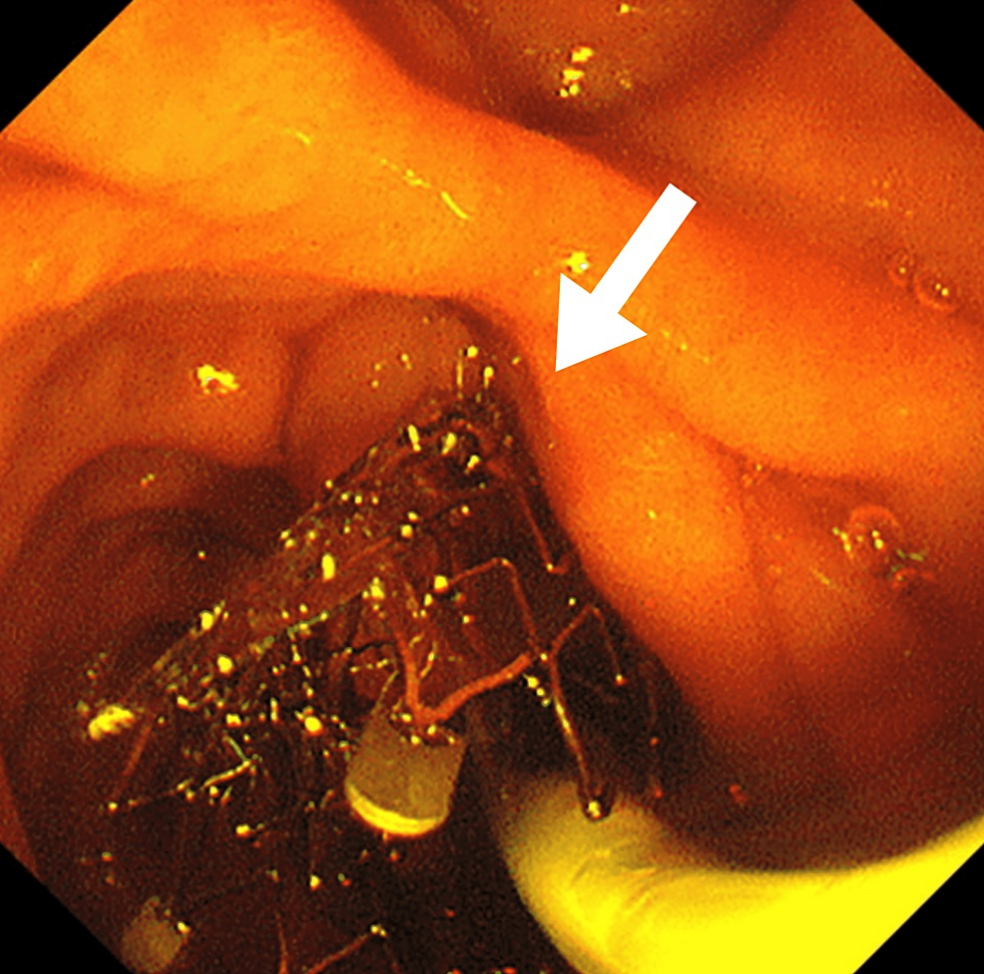
A metal stent (arrow) inserted endoscopically.

**Figure 4:**
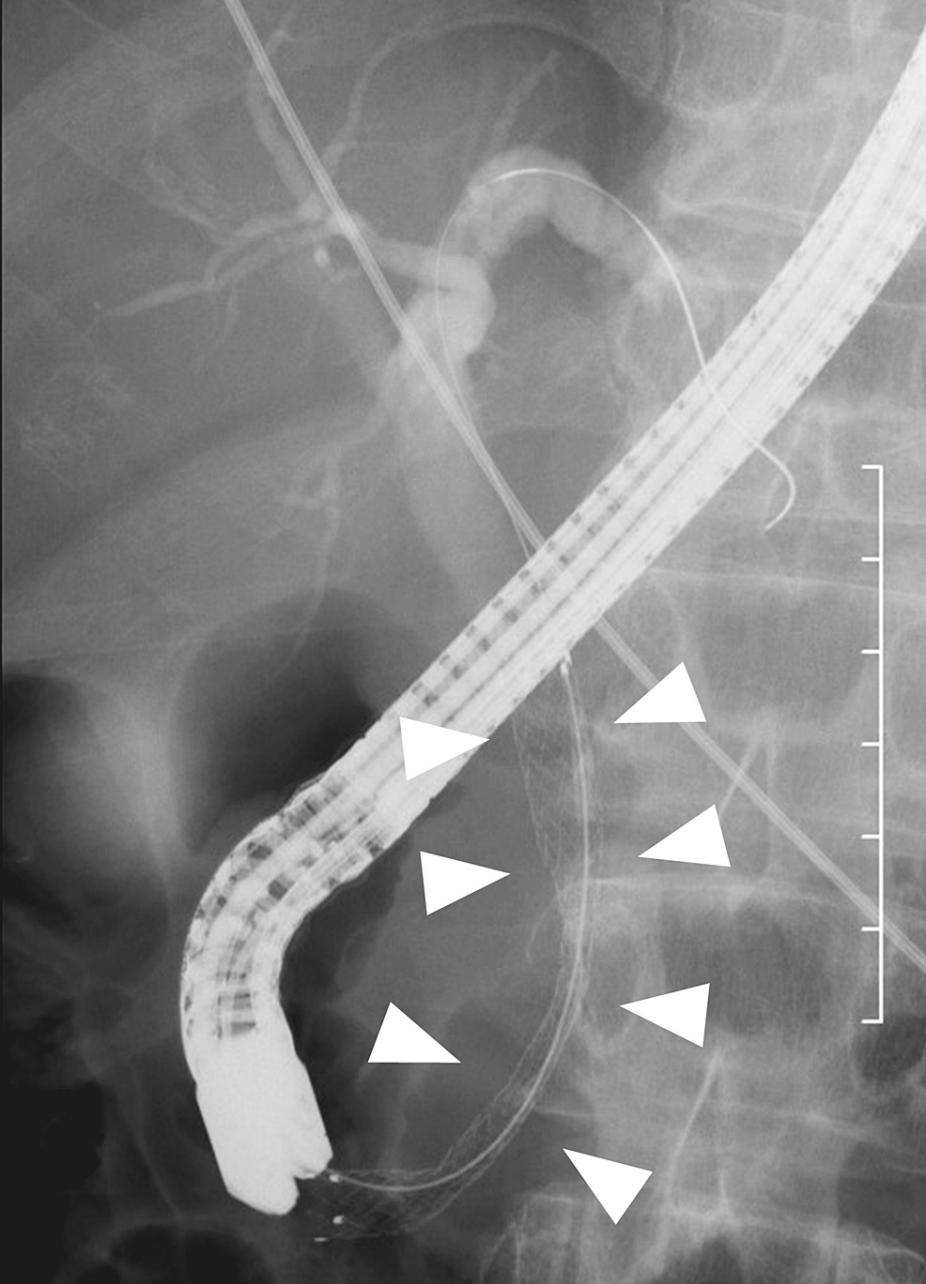
The stent (arrowhead) insertion was confirmed.

A follow-up enhanced CT scan revealed a metastatic tumor in the pancreas. Jaundice appeared during the additional four courses of chemotherapy with reduced AMR but improved with each course; however, after 12 weeks, the tumor increased markedly, and the patient complained of worsened jaundice (Figure 1). Laboratory examinations revealed elevated levels of alkaline phosphatase (241U/L), gamma GT (288U/L), and hyperbilirubinemia (toral bilirubin:4.62 mg/dL, direct bilirubin:2.83 mg/dL). Serum amylase and lipase levels were normal. Endoscopic retrograde cholangiopancreatography revealed a markedly dilated common bile duct and intrahepatic biliary duct (Figure 2). A metal stent was inserted via endoscopy, and the obstructive jaundice successfully disappeared (Figures 3-4). However, with an additional 25.8 Gy/6 fraction of palliative radiation on the pancreas, multiple brain stem metastases were found that resulted in gait disturbance and a consciousness disorder (Glasgow Coma Scale: E4V4M6). Steroid, mannitol, and 30 Gy/10 fractions of whole-brain irradiation were performed for the brain metastases. These treatments stopped the growth of the brain metastases but it did not improve his impaired consciousness. Unfortunately, the patient became bedridden and did not receive subsequent chemotherapy. He died three months after receiving home palliative care, without jaundice.

## Discussion

SNEC metastasis to the pancreas occurs in the late stage and is associated with a poor prognosis; therefore, few physicians have paid attention to and provided effective intervention for the condition [2,3].

Jaundice is the most common symptom in patients with pancreatic tumors and occurs due to compression/invasion of the bile duct in the periampullary/head mass. Pruritus, fatigue, and fat malabsorption occur in the obstructed bile ducts. Malignant bile duct obstruction may cause upper abdominal pain and severe biliary infections. It is unclear which palliative irradiation, chemotherapy, or bile duct drainage is effective for SNEC metastasis to the pancreas. If available, endoscopic biliary drainage is the best treatment option to improve jaundice and quality of life [4,5]. However, topical bypass treatment, such as biliary drainage alone, cannot control SNEC because of the variable distant metastases that occur at very high rates. Therefore, systemic chemotherapy should be restarted without an interval. Since most second-line anticancer drugs used in SCLC, such as amrubicin, irinotecan, and docetaxel, are metabolized in the liver, jaundice elimination (<2.5 mg/dL) is also required for chemotherapy. This is because unacceptable chemical toxicity can occur without the proper biliary excretion of metabolites. However, if metastasis to the pancreas causes obstructive jaundice, the antitumor effects may improve jaundice, as in this case. Liver-metabolized anticancer drugs, which are normally contraindicated, may also be worth using.

SNEC is highly radiosensitive; therefore, palliative radiation therapy for symptomatic metastatic lesions is often effective. However, radiation therapy usually takes several weeks to prepare for and execute; therefore, systemic chemotherapy may be missed for the treatment of other potential metastases. Concomitant chemoradiotherapy may be a candidate for palliative therapy.

Previous reports have successfully resected solitary pancreatic metastatic lesions from nasopharyngeal primary EPSCC [6]. However, resectable cases are extremely rare because surgery for the pancreas requires sufficient physical tolerance, and the metastatic lesions must be localized.

The choice of the approach for malignant biliary obstruction treatment is complex, and careful consideration is given to the appropriate drainage pathway, such as endoscopic or percutaneous, appropriate timing, and life expectancy of the patient [7]. This decision should be made using information from a multidisciplinary team consisting of gastroenterology, radiology, and oncology specialists.

## Conclusions

We report a very rare case of a patient with pancreatic metastasis of SNEC which is prone to distant metastasis and has a poor prognosis. Therefore, there is no adequate treatment strategy when the cancer metastasizes to rare locations. In cases of pancreatic metastasis, it is important to treat obstructive jaundice promptly and not interrupt systemic chemotherapy. Local treatment alone, such as biliary drainage, cannot control the rapid systemic distant metastasis of SNEC.
